# HIV Treatment-As-Prevention Research: Authors’ Reply

**DOI:** 10.1371/journal.pmed.1001799

**Published:** 2015-03-10

**Authors:** Till Bärnighausen, Nir Eyal, Dan Wikler

**Affiliations:** 1 Department of Global Health and Population, Harvard T.H. Chan School of Public Health, Boston, Massachusetts, United States of America; 2 Africa Centre for Health and Population Studies, University of KwaZulu-Natal, Mtubatuba, South Africa; 3 Department of Global Health and Society, Harvard Medical School, Boston, Massachusetts, United States of America

## Abstract

Till Bärnighausen and colleagues respond to comments by the HPTN 071 (PopART) Study Team, noting the distinction between the different HIV prevention questions the trial will attempt to answer.

We thank Richard Hayes and colleagues for their commentary [1] on our *PLOS Medicine* article [2]. We share the desire to learn through rigorous research whether HIV treatment-as-prevention (TasP) works in population-wide implementation. Our article pointed out that the 2013 WHO antiretroviral treatment (ART) guidelines put the on-going TasP trials at risk of failing “in their primary aim to establish the effectiveness of TasP in general populations in sub-Saharan Africa because of insufficient power” [2]. The 2013 WHO guidelines recommend substantially expanded ART eligibility. The host countries of the TasP trials are adopting the guidelines as national policy, and currently accepted ethical standards for clinical trials oblige the TasP trials to do likewise for patients enrolled in all trial arms. The problem is that the trials were designed and originally powered under the more restricted, previous ART eligibility standards and may thus become insufficiently powered to test the TasP hypothesis [2].

Hayes and colleagues assert that the HPTN071 (PopART) trial does not face this risk because “[t]he study power for the Arm A versus C comparison, the main study comparison, remains very high” when all PopART arms offer ART under the expanded eligibility standards [1]. But this comparison of Arms A versus C does not test the TasP hypothesis. Rather, it tests the very different hypothesis that an extensive HIV combination prevention package can reduce HIV incidence.

To explain, TasP aims to achieve substantial HIV incidence reductions through immediate ART initiation in all HIV-infected individuals [3–5]. In contrast, HIV combination prevention packages aim to achieve the same goal through implementation of many different interventions believed to be effective in preventing HIV [6–8]. PopART is to test both strategies. Our concern regards only the former; i.e., its test of TasP.

The PopART protocol published in *Trials* [9] in 2014 makes clear why PopART faces the risk of failing to test the TasP hypothesis because of insufficient power, just like the other trials. PopART was designed and powered “to detect a difference in incidence between Arms A and C (reflecting the full impact of the intervention), as well as the difference in intervention effect between Arms A and B (reflecting the additional effect of immediate HIV treatment compared with current national guidelines)” [9].

The additional effect of immediate HIV treatment (Arm A versus B) is the effect of TasP, namely, offering immediate HIV treatment when the “uptake and coverage of [other] HIV services is substantially expanded” [1]. Regarding this test, Hayes and colleagues write that “[f]ollowing adoption of 2013 guidelines, there will be a smaller number of HIV-infected individuals offered treatment in Arm A who would not be eligible for treatment if in Arm B communities, reducing the power to demonstrate a difference between Arms A and B” [1]. It is precisely this comparison that tests the TasP hypothesis in PopART and that is now threatened to fail because of insufficient power (Table 1). If WHO follows the United States [10,11] in recommending immediate ART initiation for all HIV-infected people in the 2015 WHO ART guidelines, PopART will face the even larger threat of completely losing the Arm A versus B comparison [12].

**Table 1:** The two interventions the HPTN071 (PopART) trial aims to test.

Intervention tested by PopART	Test	Interpretation of a significant difference between the two PopART arms	Effect of countries adopting the 2013 WHO ART guidelines	Effect of countries adopting policies of immediate ART initiation in all HIV-infected individuals
**An extensive HIV combination prevention package**	PopART Arm A versus C	Some subset of the interventions in the HIV combination prevention package—which may or may not include TasP—is effective in reducing HIV incidence	“The study power for the Arm A versus C comparison, the main study comparison, remains very high.” [1]	The study power for the Arm A versus C comparison would likely remain high
**HIV treatment-as-prevention (TasP)**	PopART Arm A versus B	TasP is effective in reducing HIV incidence	“…there will be a smaller number of HIV-infected individuals offered treatment in Arm A who would not be eligible for treatment if in Arm B communities, reducing the power to demonstrate a difference between Arms A and B.” [1]	Arm B would become equivalent to Arm A. From this point onward, none of the information collected in PopART would contribute to testing TasP

In contrast to the Arm A versus B comparison, the difference between Arms A and C in PopART is the effect of an extensive HIV combination prevention package (“the full intervention” [9]), which includes immediate HIV treatment but also many other interventions: male circumcision, condom promotion, home-based behavioural HIV risk-reduction counselling, home-based HIV testing and referral to HIV treatment and care, home-based screening for sexually transmitted infections and referral for treatment, home-based screening for tuberculosis and referral for treatment, home-based identification of pregnant women and encouragement to attend antenatal care, and encouragement to access prevention of mother-to-child transmission services for pregnant women who test HIV-positive [9].

If the PopART combination prevention package is shown to be effective, any subset of interventions in the package—which may or may not include TasP—could be responsible for the effect, and we cannot know which. Importantly, based on this comparison it is impossible to rule out that any significant effect is due entirely to those interventions in the package that have already been firmly established to be effective in preventing HIV, such as male circumcision [13–15]. The comparison of PopART Arm A versus C is thus not a valid test of the TasP hypothesis (Table 1).

Given our shared interest in testing TasP, we are glad to read that Hayes and colleagues broadly endorse two of our proposals: to increase the power to test the TasP hypothesis by pooling data across trials and to consider randomised stepped-wedge scale-up of TasP as an additional strategy to establish TasP effectiveness.

## Abbreviations

ART: antiretroviral treatment
PMTCT: prevention of mother-to-child transmission
TasP: treatment-as-prevention
